# 5-Meth­oxy-2-benzofuran-1(3*H*)-one

**DOI:** 10.1107/S1600536812044789

**Published:** 2012-11-07

**Authors:** Drielly A. Paixão, Silvana Guilardi, Jorge L. Pereira, Róbson R. Teixeira, Júnior F. Arantes

**Affiliations:** a Instituto de Química–UFU, Uberlândia, MG, Brazil; b Departamento de Química–UFV, Viçosa, MG, Brazil

## Abstract

In the title compound, C_9_H_8_O_3_, the mol­ecular skeleton is almost planar, with an r.m.s. deviation of 0.010 (2) Å. In the crystal, weak C—H⋯O hydrogen bonds connect the mol­ecules into a two-dimensional network parallel to the *ac* plane.

## Related literature

For the biological activity of isobenzofuran-1(3*H*)-one, see: Ma *et al.* (2012 ▶); Huang *et al.* (2012 ▶); Zhao *et al.* (2012 ▶); Arnone *et al.* (2002 ▶). For the synthesis, see: Zhang *et al.* (2009 ▶). For related structures, see: Sun *et al.* (2009 ▶); Mendenhall *et al.* (2003 ▶); Pereira *et al.* (2012 ▶).
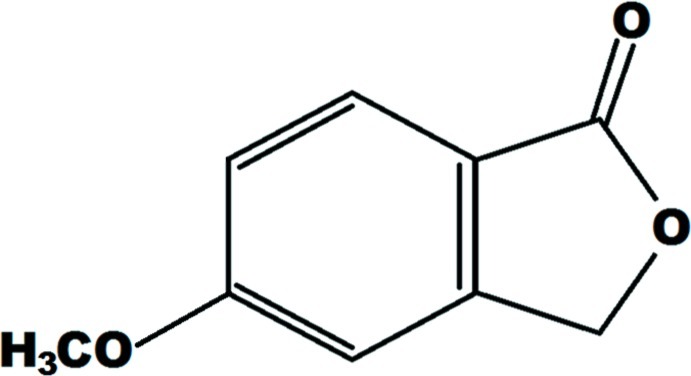


## Experimental

### 

#### Crystal data

C_9_H_8_O_3_
*M* *_r_* = 164.15
Monoclinic, 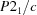

*a* = 8.1819 (9) Å
*b* = 10.4285 (18) Å
*c* = 9.2965 (9) Å
β = 99.962 (8)°
*V* = 781.26 (18) Å^3^
*Z* = 4
Mo *K*α radiation
μ = 0.11 mm^−1^
*T* = 293 K
0.30 × 0.18 × 0.16 mm

#### Data collection

Enraf–Nonius KappaCCD diffractometer
14100 measured reflections
1587 independent reflections
1101 reflections with *I* > 2σ(*I*)
*R*_int_ = 0.049

#### Refinement

*R*[*F* ^2^ > 2σ(*F* ^2^)] = 0.049
*wR*(*F* ^2^) = 0.147
*S* = 1.06
1587 reflections
109 parameters
H-atom parameters constrained
Δρ_max_ = 0.16 e Å^−3^
Δρ_min_ = −0.13 e Å^−3^

### 

Data collection: *COLLECT* (Nonius, 2000 ▶); cell refinement: *DENZO-SMN* (Otwinowski & Minor, 1997 ▶); data reduction: *DENZO-SMN*; program(s) used to solve structure: *SHELXS97* (Sheldrick, 2008 ▶); program(s) used to refine structure: *SHELXL97* (Sheldrick, 2008 ▶); molecular graphics: *ORTEP-3 for Windows* (Farrugia, 1997 ▶); software used to prepare material for publication: *WinGX* (Farrugia, 1999 ▶).

## Supplementary Material

Crystal structure: contains datablock(s) I, global. DOI: 10.1107/S1600536812044789/zs2239sup1.cif

Structure factors: contains datablock(s) I. DOI: 10.1107/S1600536812044789/zs2239Isup2.hkl

Supplementary material file. DOI: 10.1107/S1600536812044789/zs2239Isup3.cml

Additional supplementary materials:  crystallographic information; 3D view; checkCIF report

## Figures and Tables

**Table 1:** Hydrogen-bond geometry (Å, °)

*D*—H⋯*A*	*D*—H	H⋯*A*	*D*⋯*A*	*D*—H⋯*A*
C6—H6⋯O1^i^	0.93	2.54	3.419 (2)	157
C8—H8*A*⋯O2^ii^	0.97	2.52	3.372 (2)	146

Symmetry codes: (i) 
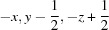
; (ii) 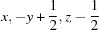.

**Fig. 1.**
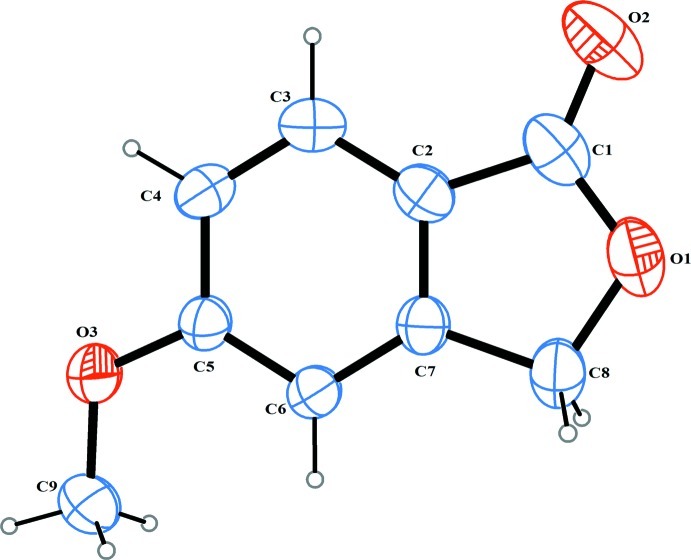
The molecular structure of the title compound, showing the atom labeling and displacement ellipsoids are drawn at the 30% probability level.

**Fig. 2.**
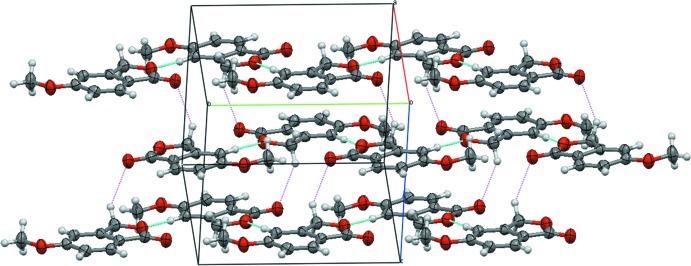
A packing diagram of the title compound. Hydrogen bonds are shown as dashed lines.

## supplementary crystallographic information

### Comment

Isobenzofuran-1(*3H*)-ones (phthalides) are a class of heterocyclic compounds which occur in several natural products and have been investigated for several biological properties, such as antiplatelet (Ma *et al.*, 2012) and antioxidant activities (Huang *et al.*, 2012), inhibition of glutamate induced cytotoxicity in PC12 cells (Zhao *et al.*, 2012) and phytotoxicity (Arnone *et al.*, 2002). The title compound, C_9_H_8_O_3_ was obtained as an intermediate in a synthetic route in the preparation of compounds endowed with phytotoxic activity and we report the crystal structure of it in a continuation of our work on the synthesis of phthalides (Pereira *et al.*, 2012).

The title molecule (Fig. 1) is essentially planar with a mean deviation of 0.010 (2) Å from the least squares plane traced by 12 non-H atoms. All bond distances and angles agree well with those reported in the related compounds (Sun *et al.*, 2009; Mendenhall *et al.*, 2003; Pereira *et al.*, 2012). In the crystal, molecules are linked *via* weak C6—H6···O1 hydrogen bonds (Table 1) forming chains along the *ac* plane. These layers are extended by C8—H8A···O2 hydrogen bonds into a two-dimensional network structure (Fig. 2).

### Experimental

Starting materials were commercially available from Sigma Aldrich (USA) and were used without further purification. 5-Methoxyisobenzofuran-1(3*H*)-one was prepared as follows (Zhang *et al.*, 2009). A tube of 40 ml equipped with a magnetic stir bar was charged with palladium(II) acetate (67.3 mg, 0.30 mmol), potassium bicarbonate (750 mg, 7.50 mmol), 4-methoxybenzoic acid (456 mg, 3.00 mmol) and dibromomethane (12 ml). The tube was sealed with a teflon cap and the reaction mixture was stirred at 140 °C for 18 h. After this time, the mixture was filtered through a pad of celite. The filtrate was concentrated under reduced pressure and the residue was purified by silica gel column chromatography eluted with hexane: ethyl acetate (2:1 *v*/*v*) to afford 5-methoxyisobenzofuran-1(3*H*)-one in 33% yield (164 mg, 1.00 mmol). The crystals suitable for X-ray crystallographic analysis were obtained by slow evaporation from acetone solution at room temperature as a yellow solid; m.p. 113.4–114.7 °C. IR (selected bands, cm^-1^): 3032, 2922, 2852, 1736, 1601, 1489, 1452, 1361, 1333, 1261, 1146, 1036, 986, 773. ^1^H NMR (300 MHz, CDCl_3_): δ 3.89 (s, 3H, H9), 5.25 (s, 2H, H8), 6.91 (d, 1H, J = 0.6 Hz, H6), 7.02 (dd, 1H, J = 8.4, 0.6 Hz, H4), 7.80 (d, 1H, J = 8.4 Hz, H3). ^13^C NMR (75 MHz, CDCl_3_): δ 56.1 (C9), 69.3 (C8), 106.2 (C6), 116.7 (C4), 118.2 (C2), 127.4 (C3), 149.6 (C7), 164.9 (C5), 171.1 (C1). HREIMS *m/z* (*M*+H^+^): Calcd for C_9_H_8_O_3_, 165.0552; found: 165.0606.

### Refinement

Hydrogen atoms were included in the refinement at calculated positions (C—H = 0.93–0.98 Å), with *U*_iso_(H) = 1.2*U*_eq_(C)(aromatic and methylene) or 1.5*U*_eq_(C)(methyl), using a riding-model approximation.

### Crystal data

**Table:**

C_9_H_8_O_3_	*D*_x_ = 1.396 Mg m^−^^3^
*M**_r_* = 164.15	Melting point = 386.4–386.7 K
Monoclinic, *P*2_1_/*c*	Mo *K*α radiation, λ = 0.71073 Å
*a* = 8.1819 (9) Å	Cell parameters from 1685 reflections
*b* = 10.4285 (18) Å	θ = 3.2–26.4°
*c* = 9.2965 (9) Å	µ = 0.11 mm^−^^1^
β = 99.962 (8)°	*T* = 293 K
*V* = 781.26 (18) Å^3^	Prism, yellow
*Z* = 4	0.30 × 0.18 × 0.16 mm
*F*(000) = 344	

### Data collection

**Table:**

Enraf–Nonius KappaCCD diffractometer	1101 reflections with *I* > 2σ(*I*)
Radiation source: Enraf Nonius FR590 X-ray source	*R*_int_ = 0.049
Graphite monochromator	θ_max_ = 26.4°, θ_min_ = 3.2°
Detector resolution: 9 pixels mm^-1^	*h* = 0→10
CCD rotation images, thick slices scans	*k* = 0→13
14100 measured reflections	*l* = −11→11
1587 independent reflections	

### Refinement

**Table:**

Refinement on *F*^2^	Primary atom site location: structure-invariant direct methods
Least-squares matrix: full	Secondary atom site location: difference Fourier map
*R*[*F*^2^ > 2σ(*F*^2^)] = 0.049	Hydrogen site location: inferred from neighbouring sites
*wR*(*F*^2^) = 0.147	H-atom parameters constrained
*S* = 1.06	*w* = 1/[σ^2^(*F*_o_^2^) + (0.0818*P*)^2^ + 0.0576*P*] where *P* = (*F*_o_^2^ + 2*F*_c_^2^)/3
1587 reflections	(Δ/σ)_max_ < 0.001
109 parameters	Δρ_max_ = 0.16 e Å^−^^3^
0 restraints	Δρ_min_ = −0.13 e Å^−^^3^

### Special details

**Table:**

Geometry. All e.s.d.'s (except the e.s.d. in the dihedral angle between two l.s. planes) are estimated using the full covariance matrix. The cell e.s.d.'s are taken into account individually in the estimation of e.s.d.'s in distances, angles and torsion angles; correlations between e.s.d.'s in cell parameters are only used when they are defined by crystal symmetry. An approximate (isotropic) treatment of cell e.s.d.'s is used for estimating e.s.d.'s involving l.s. planes.
Refinement. Refinement of *F*^2^ against ALL reflections. The weighted *R*-factor *wR* and goodness of fit *S* are based on *F*^2^, conventional *R*-factors *R* are based on *F*, with *F* set to zero for negative *F*^2^. The threshold expression of *F*^2^ > σ(*F*^2^) is used only for calculating *R*-factors(gt) *etc*. and is not relevant to the choice of reflections for refinement. *R*-factors based on *F*^2^ are statistically about twice as large as those based on *F*, and *R*- factors based on ALL data will be even larger.

### Fractional atomic coordinates and isotropic or equivalent isotropic displacement parameters (Å^2^)

**Table:**

	*x*	*y*	*z*	*U*_iso_*/*U*_eq_	
O3	0.37522 (15)	−0.24147 (11)	0.61307 (13)	0.0781 (4)	
O1	0.05757 (18)	0.21719 (13)	0.30246 (15)	0.0903 (5)	
C7	0.16866 (17)	0.02605 (15)	0.40441 (16)	0.0577 (4)	
C5	0.32245 (19)	−0.12294 (14)	0.56458 (16)	0.0591 (4)	
C3	0.3507 (2)	0.10279 (17)	0.61788 (19)	0.0691 (5)	
H3	0.3971	0.17	0.6769	0.083*	
C6	0.20840 (18)	−0.10008 (15)	0.43826 (16)	0.0578 (4)	
H6	0.1609	−0.1669	0.3791	0.069*	
O2	0.1929 (2)	0.35524 (13)	0.46580 (19)	0.1117 (6)	
C4	0.39278 (19)	−0.02130 (16)	0.65283 (17)	0.0662 (4)	
H4	0.4694	−0.0388	0.7367	0.079*	
C2	0.2366 (2)	0.12584 (14)	0.49162 (18)	0.0631 (4)	
C8	0.0518 (2)	0.07991 (17)	0.27798 (19)	0.0757 (5)	
H8A	0.0874	0.0587	0.1866	0.091*	
H8B	−0.0595	0.0471	0.2757	0.091*	
C9	0.3066 (3)	−0.34911 (17)	0.5294 (2)	0.0956 (7)	
H9A	0.3534	−0.4266	0.5748	0.143*	
H9B	0.3318	−0.3432	0.4325	0.143*	
H9C	0.1884	−0.3501	0.5244	0.143*	
C1	0.1678 (3)	0.24532 (17)	0.4269 (2)	0.0804 (6)	

### Atomic displacement parameters (Å^2^)

**Table:**

	*U*^11^	*U*^22^	*U*^33^	*U*^12^	*U*^13^	*U*^23^
O3	0.0902 (8)	0.0618 (7)	0.0735 (7)	0.0057 (6)	−0.0102 (6)	0.0080 (5)
O1	0.1053 (10)	0.0749 (9)	0.0911 (9)	0.0277 (7)	0.0182 (8)	0.0207 (7)
C7	0.0555 (8)	0.0608 (9)	0.0574 (8)	0.0033 (6)	0.0117 (6)	0.0053 (6)
C5	0.0602 (8)	0.0573 (10)	0.0576 (8)	0.0000 (6)	0.0046 (7)	0.0042 (6)
C3	0.0743 (10)	0.0658 (10)	0.0678 (10)	−0.0129 (8)	0.0144 (8)	−0.0104 (8)
C6	0.0582 (8)	0.0576 (9)	0.0555 (8)	−0.0025 (6)	0.0038 (6)	−0.0010 (6)
O2	0.1675 (16)	0.0565 (9)	0.1221 (12)	0.0114 (8)	0.0560 (12)	0.0022 (7)
C4	0.0636 (9)	0.0742 (11)	0.0575 (8)	−0.0091 (7)	0.0008 (7)	−0.0032 (7)
C2	0.0676 (9)	0.0564 (10)	0.0687 (10)	−0.0011 (6)	0.0210 (8)	−0.0008 (7)
C8	0.0778 (11)	0.0768 (12)	0.0706 (10)	0.0153 (9)	0.0074 (8)	0.0127 (8)
C9	0.1225 (17)	0.0552 (11)	0.0988 (14)	0.0070 (10)	−0.0099 (12)	−0.0016 (9)
C1	0.1003 (14)	0.0612 (11)	0.0877 (13)	0.0118 (9)	0.0391 (11)	0.0066 (9)

### Geometric parameters (Å, º)

**Table:**

O3—C5	1.3603 (18)	C3—H3	0.93
O3—C9	1.425 (2)	C6—H6	0.93
O1—C1	1.370 (3)	O2—C1	1.209 (2)
O1—C8	1.449 (2)	C4—H4	0.93
C7—C2	1.376 (2)	C2—C1	1.454 (2)
C7—C6	1.378 (2)	C8—H8A	0.97
C7—C8	1.491 (2)	C8—H8B	0.97
C5—C6	1.388 (2)	C9—H9A	0.96
C5—C4	1.402 (2)	C9—H9B	0.96
C3—C4	1.364 (2)	C9—H9C	0.96
C3—C2	1.388 (2)		
			
C5—O3—C9	117.55 (14)	C7—C2—C1	108.43 (16)
C1—O1—C8	110.05 (13)	C3—C2—C1	130.82 (16)
C2—C7—C6	122.13 (14)	O1—C8—C7	104.45 (14)
C2—C7—C8	108.56 (14)	O1—C8—H8A	110.9
C6—C7—C8	129.31 (14)	C7—C8—H8A	110.9
O3—C5—C6	124.38 (14)	O1—C8—H8B	110.9
O3—C5—C4	114.75 (14)	C7—C8—H8B	110.9
C6—C5—C4	120.87 (15)	H8A—C8—H8B	108.9
C4—C3—C2	118.08 (15)	O3—C9—H9A	109.5
C4—C3—H3	121	O3—C9—H9B	109.5
C2—C3—H3	121	H9A—C9—H9B	109.5
C7—C6—C5	117.03 (14)	O3—C9—H9C	109.5
C7—C6—H6	121.5	H9A—C9—H9C	109.5
C5—C6—H6	121.5	H9B—C9—H9C	109.5
C3—C4—C5	121.14 (15)	O2—C1—O1	120.56 (18)
C3—C4—H4	119.4	O2—C1—C2	131.0 (2)
C5—C4—H4	119.4	O1—C1—C2	108.49 (15)
C7—C2—C3	120.75 (15)		
			
C9—O3—C5—C6	−1.0 (2)	C8—C7—C2—C1	0.17 (17)
C9—O3—C5—C4	179.11 (15)	C4—C3—C2—C7	−0.2 (2)
C2—C7—C6—C5	−0.6 (2)	C4—C3—C2—C1	179.72 (15)
C8—C7—C6—C5	−179.90 (15)	C1—O1—C8—C7	1.42 (18)
O3—C5—C6—C7	−179.79 (13)	C2—C7—C8—O1	−0.95 (16)
C4—C5—C6—C7	0.1 (2)	C6—C7—C8—O1	178.38 (14)
C2—C3—C4—C5	−0.3 (2)	C8—O1—C1—O2	178.93 (16)
O3—C5—C4—C3	−179.70 (13)	C8—O1—C1—C2	−1.36 (19)
C6—C5—C4—C3	0.4 (2)	C7—C2—C1—O2	−179.60 (18)
C6—C7—C2—C3	0.7 (2)	C3—C2—C1—O2	0.5 (3)
C8—C7—C2—C3	−179.91 (15)	C7—C2—C1—O1	0.73 (19)
C6—C7—C2—C1	−179.22 (13)	C3—C2—C1—O1	−179.17 (16)

### Hydrogen-bond geometry (Å, º)

**Table:**

*D*—H···*A*	*D*—H	H···*A*	*D*···*A*	*D*—H···*A*
C6—H6···O1^i^	0.93	2.54	3.419 (2)	157
C8—H8*A*···O2^ii^	0.97	2.52	3.372 (2)	146

Symmetry codes: (i) −*x*, *y*−1/2, −*z*+1/2; (ii) *x*, −*y*+1/2, *z*−1/2.
